# Breast and ovarian cancer penetrance of *BRCA1/2* mutations among Hong Kong women

**DOI:** 10.18632/oncotarget.24382

**Published:** 2018-02-02

**Authors:** LingJiao Zhang, Vivian Y. Shin, Xinglei Chai, Alan Zhang, Tsun L. Chan, Edmond S. Ma, Timothy R. Rebbeck, Jinbo Chen, Ava Kwong

**Affiliations:** ^1^ Department of Biostatistics, Epidemiology, and Informatics, University of Pennsylvania Perelman School of Medicine, Philadelphia, PA, USA; ^2^ Department of Surgery, the University of Hong Kong, Hong Kong; ^3^ Sidwell High School, Bethesda, MD, USA; ^4^ Department of Molecular Pathology, Hong Kong Sanatorium & Hospital, Hong Kong; ^5^ Hong Kong Hereditary Breast Cancer Family Registry, Hong Kong; ^6^ Dana Farber Cancer Institute and Harvard TH Chan School of Public Health, Boston, MA, USA; ^7^ Department of Surgery, Hong Kong Sanatorium & Hospital, Hong Kong

**Keywords:** BRCA1, BRCA2, breast and ovarian cancer, penetrance, Chinese population

## Abstract

Germline mutations in *BRCA1* and *BRCA2* (*BRCA1/2*) are associated with increased risk of breast and ovarian cancer. The penetrance of breast and ovarian cancer in *BRCA1/2* mutation carriers has been well characterized in Caucasian but not in Asian. Two studies have investigated the breast cancer risk in Asian women with *BRCA1/2* mutations, and no published estimates are available for ovarian cancer. Therefore, we estimated the age-specific cumulative risk of *BRCA1/2*-associated breast and ovarian cancer in Chinese women. From Jan 2007 to Nov 2015, the Hong Kong Hereditary Breast Cancer Family Registry identified 1635 families with hereditary breast-ovarian cancer. Among probands in these families, 66 had *BRCA1* mutations, 84 had *BRCA2* mutations, and 1,485 tested negative for *BRCA1/2* mutations. Using the female first-degree relatives of these probands, we estimated the risk of breast and ovarian cancer using a modified marginal likelihood approach. Estimates of breast cancer penetrance by age 70 were 53.7% (95% CI 34.5-71.6%) for *BRCA1* mutation carriers and 48.3% (95% CI 31.8-68.5%) for *BRCA2*. The estimated risk of ovarian cancer by age 70 was 21.5% and 7.3% for Chinese women carrying *BRCA1* or *BRCA2* mutation respectively. A meta-analysis of available studies in Asian women revealed pooled estimates of breast cancer risk by age 70 of 44.8% (95% CI 33-57.2%) and 40.7% (95% CI 31.3-50.9%) for *BRCA1* and *BRCA2* mutation carriers respectively. These data suggest that *BRCA1/2*-associated breast cancer risk for Chinese women is similar to that for Caucasian women, although *BRCA1/2*-associated ovarian cancer risks are lower for Chinese women.

## INTRODUCTION

*BRCA1* and *BRCA2* (*BRCA1/2*) are human tumor suppressor genes which play a role in DNA damage repair and transcriptional regulation [1]. Germline mutations in *BRCA1/2* are associated with an increased risk and early age onset for hereditary breast and ovarian cancer [2]. Genetic counseling is now routinely offered to individuals with high-risk of carrying a *BRCA1* or *BRCA2* mutation. Correspondingly, personalized prevention strategies will be offered according to their risk level, which is often assessed through a risk prediction model. Thus, risk assessment in *BRCA1/2* mutation carriers is of great importance in clinical service and cancer management.

The risk estimates of breast and ovarian cancer in *BRCA1/2* mutation carriers have been well characterized for Caucasian women and individuals of Ashkenazi-Jewish background [2–4]. According to the most recent meta-analysis, the cumulative breast cancer risk by age 70 years was estimated to be 55% (95% CI: 50-59%) and 47% (95% CI: 42-51%) for Caucasian women carrying a *BRCA1* or *BRCA2* mutation respectively. The estimates of ovarian cancer penetrance by age 70 were 39% (95% CI: 34-45%) for *BRCA1* mutation carriers and 17% (95% CI: 13- 21%) for *BRCA2* mutation carriers. Since the mutation frequency and cancer incidence vary by ethnic groups [5, 6], these estimates cannot be extrapolated to a large outbred population such as East Asia.

The contribution of *BRCA1* and *BRCA2* mutations to breast and ovarian cancer incidence has not been well explored in Asian population, where both cancer incidence and mutation prevalence are lower compared to western countries [7, 8]. So far only two studies have investigated breast cancer risk in Asian women with *BRCA1*/2 mutations. Yao et al. identified 70 *BRCA1* and 55 *BRCA2* mutation-carrying families from 1,816 unselected Chinese women with breast cancer, and estimated the breast cancer risk by age 70 as 37.9% (95% CI: 24.1-54.4%) for *BRCA1* and 36.5% (95% CI: 26.7-51.8%) for *BRCA2* using a kin-cohort design [9]. Park et al. reported their estimates for Korean women based on 151 *BRCA1* and 225 *BRCA2* mutation-carrying families using a modified segregation analysis [10]. According to their study, Korean women with *BRCA1* mutation had a cumulative risk of 49% (95% CI: 11-98%) for development of breast cancer by age 70, and the risk was 35% (95% CI: 16-65%) for *BRCA2*. However, there is no published ovarian cancer estimates associated with these mutations in Asian women.

The aim of our study is to estimate breast and ovarian cancer risk in Chinese female *BRCA1*/*2* mutation carriers using a more efficient method based on a relatively large study population. We will also conduct a meta-analysis to integrate available estimates of breast cancer risk in Asian women carrying a *BRCA1* or *BRCA2* germline mutation into a consensus estimate of penetrance.

## RESULTS

A total of 66 *BRCA1*, 84 *BRCA2*, and 1,485 mutation-negative families were included in our study. The mutation frequencies in this sample of high-risk families for *BRCA1* and *BRCA2* were estimated as 2.1% and 2.7% respectively. Data for the 5,949 first-degree relatives was utilized to obtain estimates of breast cancer penetrance in mutation carriers (230 from *BRCA1* carrier relatives, 309 from *BRCA2* carrier relatives and 5,410 from non-carrier relatives). Although most of the first-degree relatives were un-genotyped (67.4%, 73.1%, and 99.6% for *BRCA1, BRCA2* and non-carrier relatives respectively), 16.1% and 14.6% of first-degree relatives were confirmed mutation carriers for *BRCA1* and *BRCA2* relatives respectively, and 16.5% and 12.3% were confirmed non-carriers.

### Risk of breast cancer in first-degree relatives

A total of 568 breast cancer cases occurred in first-degree relatives, with an incidence of 21.3%, 20.4, and 8.4% for *BRCA1*, *BRCA2* and non-carrier families, respectively (Table 1). The risk of breast cancer in the first-degree relatives of *BRCA1* and *BRCA2* probands was significantly higher than in first-degree relatives of non-carrier families (hazard ratio, HR=3.31, 95% CI 2.46-4.44, *p*<0.001; HR=3.31, 95% CI 2.46-4.44, *p*<0.001 for *BRCA1* and *BRCA2* respectively). For probands, the mean age at diagnosis of breast cancer in *BRCA1* carriers was significantly earlier than non-carrier (41.8 vs 44.8 years, *p*=0.02). There was no significant difference in age at diagnosis between *BRCA1* and *BRCA2* carrier families (41.8 vs 43.4 years, *p*=0.28) nor *BRCA2* carrier families and non-carrier families (43.4 vs 44.8 years, *p*=0.22). The results were similar for the first-degree relatives. The mean age at diagnosis in first-degree relatives of *BRCA1* carriers or *BRCA2* carriers was significantly earlier than that of non-carrier families (45.1 vs 51.1 years, p=0.0005; 45.8 vs 51.1 years, p=0.0005). There was no significant difference between *BRCA1* and *BRCA2* families (45.1 vs 45.8 years, p=0.76).

**Table 1:** Number and age at diagnosis of breast and ovarian cancer cases in *BRCA1*, *BRCA2*, and non-carrier probands and first-degree relatives

	No. breast cancer	Age at diagnosis	Age at diagnosis	No. ovarian cancer	Age at diagnosis	Age at diagnosis
	No. (%)	Mean (SE)	Median (IQR)	No. (%)	Mean (SE)	Median (IQR)
**Probands**						
** *BRCA1***	58 (87.9)	41.8 (1.2)	40 (35-48)	17 (25.8)	47.1 (2.0)	45 (41-52)
** *BRCA2***	83 (98.8)	43.4 (0.9)	42 (37-49)	4 (4.8)	46.5 (0.9)	46 (45.5-47.5)
** Non-carrier**	1457 (98.1)	44.8 (0.3)	44 (38-50)	35 (2.4)	40.6 (1.9)	43 (33-49)
**First-Degree Relatives with known *BRCA* status**						
** *BRCA1***	49 (21.3)	45.1 (1.8)	42 (36-53)	20 (8.7)	50.4 (1.6)	50 (45-55)
** *BRCA2***	63 (20.4)	45.8 (1.5)	45 (38-50)	5 (1.6)	66.0 (4.8)	62 (61-69)
** Non-carrier**	456 (8.4)	51.1 (0.5)	50 (44-58)	42 (0.8)	49.0 (2.4)	49 (35-60)

IQR: Interquartile range.

### Age-specific penetrance of breast cancer in mutation carriers

**Figure 1:**
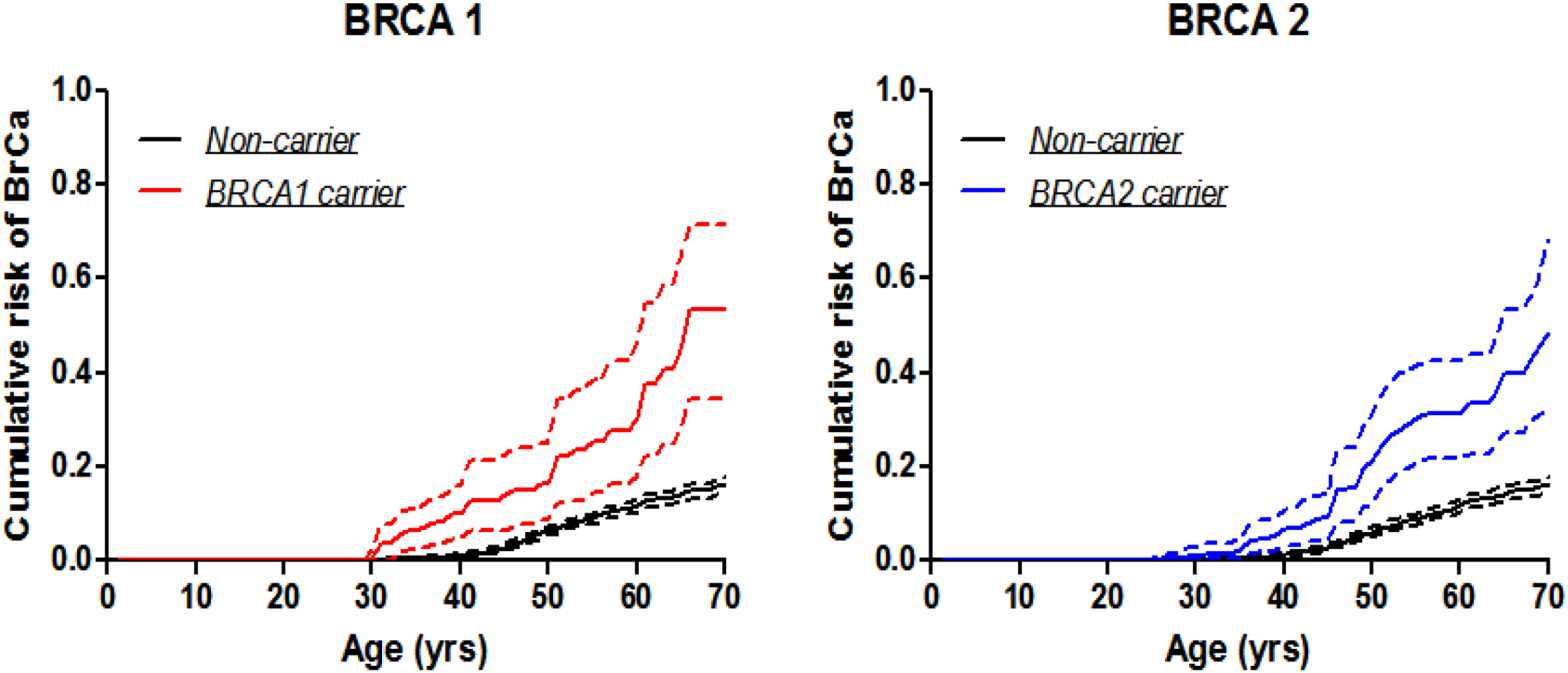
Breast cancer penetrance estimates by the modified kin-cohort method using data from first-degree relatives of probands who carry *BRCA1* or *BRCA2* mutations from Hong Kong Hereditary and High Risk Breast Cancer Programme

The estimated penetrance of breast cancer in *BRCA1* mutation carriers by age 70 years was 53.7% (95% CI: 34.5-71.6%) (Table 2 & Figure 1), which is higher than that for Korean women reported by Park et al., 49% (95% CI: 11-98%) [10], and for Chinese women reported by Yao et al., 37.9% (95% CI: 24.1-54.4%) [9]. Estimates by age 40, 50, 60, 70 years from all three studies were shown in Table 3. The meta-analytic estimate of penetrance by age 70 years based on these three studies was 44.8% (95% CI: 33-57.2%) (Test for heterogeneity: p=0.49). The meta-analytic estimates were 5.2% (95% CI: 1.3-18.2%), 13.6% (95% CI: 8.9-20.3%), and 30.9% (95% CI: 21.5-42.2%) for age intervals 20 to 40, 20 to 50, and 20 to 60 years, respectively.

**Table 2:** Age-specific penetrance of breast cancer (×100) by *BRCA1* and *BRCA2* mutation status, estimated using data from the first-degree relatives by the modified kin-cohort method

	*BRCA1* carrier	*BRCA2* carrier	Non-carrier
Age Interval	Penetrance (95% CI)	Penetrance (%) (95% CI)	Penetrance (%) (95% CI)
**20-40 years**	10.0 (4.7-15.7)	6.7 (3.2-11.0)	1.1 (0.8-1.5)
**20-50 years**	16.3 (8.6-25.0)	21.1 (12.3-31.1)	6.3 (5.4-7.2)
**20-60 years**	30.5 (18.0-46.6)	31.4 (21.6-42.6)	11.8 (10.5-13.0)
**20-70 years**	53.7 (34.5-71.6)	48.3 (31.8-68.5)	16.1 (14.3-17.8)

**Table 3:** Meta-analytic estimates of breast cancer penetrance (× 100) in Asian women who carry *BRCA1* or *BRCA2* mutations

Study	Korea	Beijing	Hong Kong	Meta-analytic Estimates
Age Interval	Penetrance (%) (95% CI)	Penetrance (%) (95% CI)	Penetrance (%) (95% CI)	Penetrance (%) (95% CI)
***BRCA1***				
20-40 years	10 (2-45)	2.2 (0-4.4)	10.0 (4.7-15.7)	5.2 (1.3-18.2)
20-50 years	27 (5-84)	9.9 (3.3-17.2)	16.3 (8.6-25.0)	13.6 (8.9-20.3)
20-60 years	41 (9-95)	29.3 (17.6-47.1)	30.5 (18.0-46.6)	30.9 (21.5-42.2)
20-70 years	49 (11-98)	37.9 (24.1-54.4)	53.7 (34.5-71.6)	44.8 (33.0-57.2)
***BRCA2***				
20-40 years	6 (3-15)	1.2 (0-3.1)	6.7 (3.2-11.0)	4.5 (1.8-10.7)
20-50 years	18 (8-39)	10.7 (9.6-16.9)	21.1 (12.3-31.1)	15.8 (10.1-24.1)
20-60 years	28 (13-56)	27.2 (19.0-38.5)	31.4 (21.6-42.6)	29.6 (23.1-36.9)
20-70 years	35 (16-65)	36.5 (26.7-51.8)	48.3 (31.8-68.5)	40.7 (31.3-50.9)

Similarly, for *BRCA2* mutation carriers, the estimated penetrance of breast cancer by age 70 years was 48.3% (95% CI: 31.8-68.5%) (Table 2 & Figure 1), which is similar to Korean women, 35% (95% CI: 16-65%)[10] and Chinese women in Beijing, 36.5% (95% CI: 26.7-51.8%) [9] (Table 3). The meta-analytic estimate of penetrance by age 70 years based on these three studies was 40.7% (95% CI: 31.3-50.9%) (Test for heterogeneity: p=0.16). The meta-analytic estimates were 4.5% (95% CI: 1.8-10.7%), 15.8% (95% CI: 10.1-24.1%), and 29.6% (95% CI: 23.1-36.9%) for age intervals 20 to 40, 20 to 50, and 20 to 60 years, respectively.

### Age-specific penetrance for ovarian cancer

**Figure 2:**
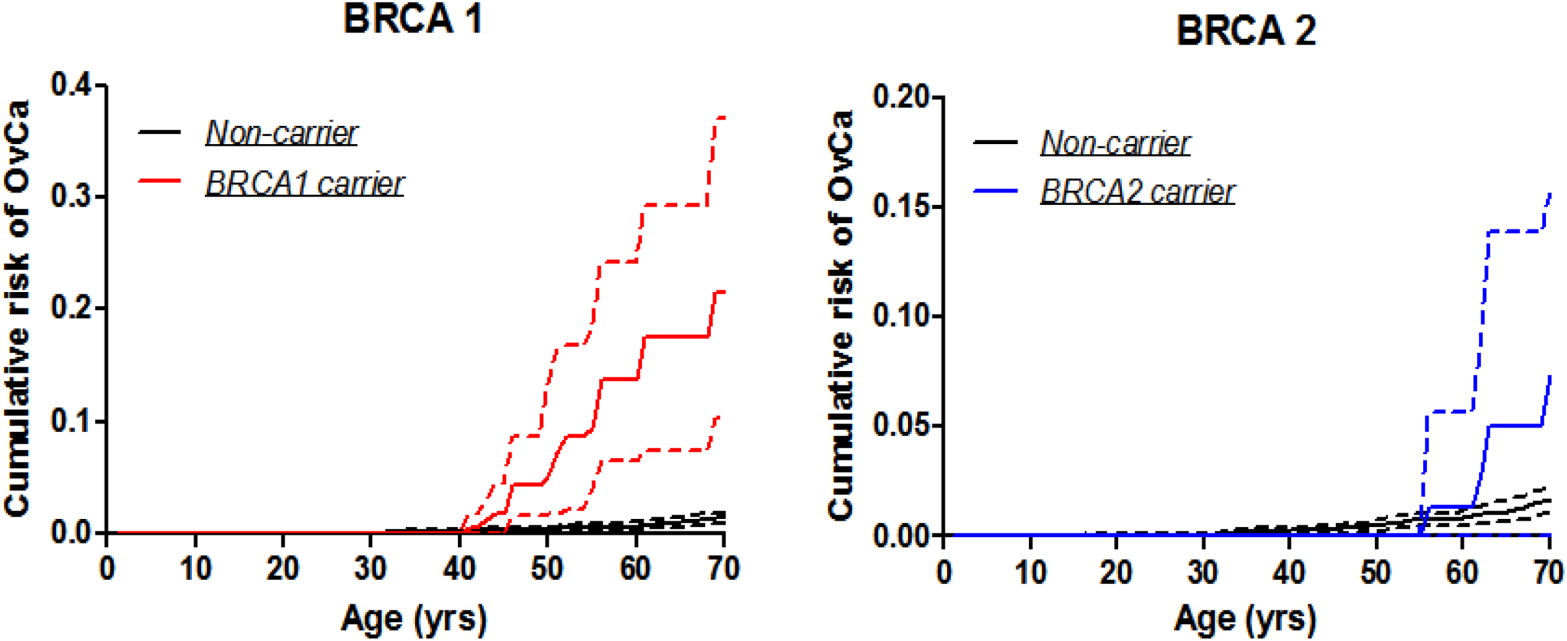
Ovarian cancer penetrance estimates by the modified kin-cohort method using data from first-degree relatives of probands who carry *BRCA1* or *BRCA2* mutations from Hong Kong Hereditary and High Risk Breast Cancer Programme

The estimated penetrance of ovarian cancer by age 70 years was 21.5% (95% CI: 10.4-37.0%) for *BRCA1* mutation carriers and 7.3% for *BRCA2* mutation carriers. The penetrance estimate by age 60 years was 13.7% (95% CI 6.6-24.3%) for *BRCA1* mutation carriers and 1.3% for *BRCA2* mutation carriers (Table 4 & Figure 2). The number of ovarian cancer cases among *BRCA2* carriers was too small to permit reasonable estimation of confidence intervals.

**Table 4:** Age-specific penetrance of ovarian cancer (× 100) by *BRCA1* and *BRCA2* mutation status, estimated using data from the first-degree relatives by the modified kin-cohort method

	*BRCA1* carrier	*BRCA2* carrier	Non-carrier
Age Interval	Penetrance (%) (95% CI)	Penetrance (%) (95% CI)	Penetrance (%) (95% CI)
**30-50 years**	5.0 (1.8-13.3)	-	0.4 (0.2-0.6)
**30-60 years**	13.7 (6.6-24.3)	1.3^1^	0.7 (0.4-1.0)
**30-70 years**	21.5 (10.4-37.1)	7.3^1^	1.4 (0.9-1.9)

^1^Number of events too small to allow for reasonable estimates of confidence intervals.

## DISCUSSION

*BRCA1*/2 mutations are known to increase the lifetime risk of breast cancer by 50-87% and ovarian cancer by 10-40%, based on data derived primarily from Caucasians [2]. *BRCA1*/2 mutation carriers have been managed based on the existing guidelines such as NCCN guidelines. These guidelines include more intensive screening, risk-reducing surgeries such as mastectomy and salpingo-oopherectomy, and even the use of specific types of chemotherapy such as platinum agents or targeted therapy such as poly (ADP-ribose) polymerase (PARP) inhibitors [15, 16]. Our current data revealed that the cumulative risks of ovarian cancer in Chinese is lower than that of the Caucasians, preventive surgery (e.g. salpingo-oopherectomy) maybe “overtreated” the patients or those healthy individuals who harbor the mutations. Hence, screening of germline *BRCA1/2* mutations is crucial for appropriate clinical management of cancer risk, yet more studies on the prevalence and cumulative breast cancer risk for these genes in Asian women are warranted.

We reported the first large-scale estimate of *BRCA1*/2 prevalence in Southern Chinese families, ascertained through patients with triple negative breast cancer and those that had a family history of breast and/or ovarian cancers. These women have an elevated chance of carrying *BRCA1*/2 mutations [11]. There was also a difference in the observed spectrum of mutations where over 40% of the *BRCA* mutations were novel and had never been reported. We previously also collected data from a *BRCA* consortium with 47 Asian countries and Asian population residing in North America on all the *BRCA* mutations in breast cancer patients [17]. The prevalence of *BRCA* mutations varies in different Asian cohorts, with 21.7% (*BRCA1* 9.3%; *BRCA2* 12.4%) in patients with family history of breast or ovarian cancer in Korea [18] and 17% (*BRCA1* 11%; *BRCA2* 6%) in Malaysian patients with early onset of breast cancer [19]. In the present study, our Southern Chinese population had a higher *BRCA1* mutation than *BRCA2* mutation than in Mainland China (*BRCA1* 10.6%; *BRCA2* 5.2%), despite there were disparities in the selection criteria in each study [20]. In Hong Kong, the prevalence of *BRCA1/2* mutations was 9.4%, and a slightly higher *BRCA2* mutation rate was observed [11]. Considering patients with triple-negative breast cancer, *BRCA1* mutation dominancy was seen in both Asian and Caucasian populations [17].

It is important to know the estimated risk for women who carry *BRCA* mutations, so that high risk patients can be identified for genetic screening and better surveillance options and treatment can be implemented in the genetic counseling. Risk prediction models such as BOADICEA, BRCAPRO and Myriad models are designed for the estimation of risk among women carrying a *BRCA* mutation in hereditary breast cancers [21, 22]. These prediction tools have been developed based on different ethnic groups and with slight difference in predictive factors. Therefore, their performance needs to be validated for the selection of high risk patients [23, 24]. In view of this, we adopted the kin-cohort approach which treated the first-degree relatives of the probands as an ascertained cohort to the present study and modified the R kin. cohort package to improve the efficiency [13]. Meta-analytic penetrance estimates of *BRCA1* and *BRCA2* mutations in Asian carriers (results from three cohort studies) were 44.8% and 40.7% respectively. In general, Hong Kong has the highest estimated risk of breast cancer by age 70 years for both *BRCA1* and *BRCA2* carriers, but still much lower than that in Caucasians (40-87% for *BRCA1* and 27-84% for *BRCA2*) [25, 26]. The cumulative estimates of ovarian cancer in Caucasians ranged from 16-68% and 11-27% for *BRCA1* and *BRCA2* carriers respectively, which are in line with our findings that *BRCA2* carriers tend to have a lower risk of ovarian cancer [25, 26]. The variability of these estimates can be influenced by other non-genetic and environmental factors (e.g. breast feeding, pregnancy and radiation exposure) [27].

To the best of our knowledge, this is the first study to report the penetrance of *BRCA* mutation carriers in the Hong Kong Chinese population with breast and ovarian cancers, which differs from that in Caucasian populations. In this study, the ovarian cancer estimates was found to be different from that of the Caucasians. The cumulative estimates of breast cancer were similar, but the ethnicity-specific estimates are more relevant in terms of clinical management and surveillance. Hence, clinicians and genetic counselors need to be cautious when quoting cancer risk estimates from the Western data in explaining genetic test results to patients. Better understanding of the difference in penetrance and cancer risks would mean that present guidelines based on Western data may need to be modified to better suit ethnicities. Nevertheless, large-scale multicenter studies in different regions of China will enhance the estimation of the overall cancer risk in *BRCA* carriers with Chinese ethnicity. Genetic counseling and testing services are not a common practice in Mainland China and other Asian countries, except for Korea, mainly because of the limited access to qualified laboratory and healthcare professionals, and also resources for laboratory and clinical services are scanty [28]. Although genetic testing is very much standard care in the West, there is still limited resource to support testing and allow more popularized genetic testing in Asia. As more prevalence and spectrum data of Asian ethnicities emerges which is found to diverge from the West, estimated penetrance from larger cohort studies is needed to better guide patient care in these high-risk cohorts.

## MATERIALS AND METHODS

### Ethics statement

The study was performed in accordance with the Declaration of Helsinki. Written informed consent was obtained from all participants recruited in this study. This study was approved by the Institutional Review Board of the University of Hong Kong/Hospital Authority West Cluster and other contributing hospitals in Hong Kong (UW-16-274 T1299).

### Study participants

In this study, a total of 1,635 Chinese families were recruited at the Hong Kong Hereditary Breast Cancer Family Registry from Jan 2007 to Nov 2015. The patient selection criteria were described previously [11]. A standard epidemiological questionnaire, including a detailed family history, was administered to patients and medical information, including pathology reports, was retrieved from the patient's medical records. Information from the epidemiological questionnaire included age at breast/ovarian cancer diagnosis, other cancers diagnosed in the patient, and a family history of breast, ovarian, and other cancers in first, second, and third degree relatives. Also, the date for preventive bilateral risk-reducing salpingo-oophorectomy, bilateral risk-reducing mastectomy, last follow-up or death were collected. This cohort of families who were previously characterized by Sanger sequencing and 1,100 probands were subjected to NGS screening. Concurrent with sequencing, all patients were tested for large genomic rearrangement of *BRCA1* and *BRCA2* by multiplex ligation-dependent probe amplification (MLPA). Characteristics of patient cohorts were shown in Table 5.

**Table 5:** Number of total and genotyped first degree relatives from the 66 *BRCA1* families, 84 *BRCA2* families, and 1485 non-carrier families

	*BRCA1*		*BRCA2*		Non-carrier	
	Total No. (%)	Per Family Median No. (IQR)	Total No. (%)	Per Family Median No. (IQR)	Total No. (%)	Per Family Median No. (IQR)
**First Degree Relatives**	230 (77.7)	3 (2-4)	309 (78.6)	3 (2-4)	5410 (78.4)	3 (2-5)
**Mother**	66 (28.7)	1 (1-1)	84 (27.2)	1 (1-1)	1485 (27.4)	1 (1-1)
**Daughter**	37 (16.1)	0 (0-1)	48 (15.5)	0 (0-1)	967 (17.9)	0 (0-1)
**Sister**	127 (55.2)	1 (1-3)	177 (57.3)	2 (1-3)	2958 (54.7)	2 (1-3)
**Carriers**	37 (16.1)	1 (1-2)	45 (14.6)	1 (1-2)	1 (<0.1)	0 (0-0)
**Non-carriers**	38 (16.5)	0 (0-1)	38 (12.3)	0 (0-1)	19 (0.4)	1 (1-1)
**Un-genotyped**	155 (67.4)	2 (1-3)	226 (73.1)	2 (1-3)	5390 (99.6)	3 (2-5)

IQR: Interquartile range.

All male relatives were excluded from the study due to gender difference in breast cancer penetrance. Only first degree relatives were included in the analysis due to lack of statistical methodology for incorporating distant relatives. Previous study showed that patients with triple negative breast cancer and those that had a family history of breast and/or ovarian cancers have increased risk of carrying *BRCA1*/2 mutations [11]. 66 female *BRCA1* carrier probands were identified, and age-at-onset data for 1,747 relatives were collected. 230 first-degree relatives were included in the analysis, with 66 mothers, 37 daughters and 127 sisters (Supplementary Figure 1). Among the 230 first-degree relatives, 37 were confirmed *BRCA1* mutation carriers, 38 non-carriers, and 155 were not tested.

Moreover, 84 female *BRCA2* carrier probands were recruited, with information on 2,330 relatives (1,145 male vs. 1,185 female) available. 309 female first-degree relatives, including 84 mothers, 48 daughters and 177 sisters, were included in the current analysis (Supplementary Figure 2). Of those, 45 were confirmed *BRCA2* mutation carriers, 38 non-carriers, and 226 were not tested. In addition, 1,485 non-carrier female probands provided information for 40,015 relatives. Of 5,410 first-degree relatives (1,485 mothers, 967 daughters, 2,958 sisters), only 1 was *BRCA2* positive, 19 were *BRCA1*/2 negative, and the rest were not tested (Supplementary Figure 3).

535 out of 7584 first degree relatives missed the time to event data (21, 22, 492 for *BRCA1*, *BRCA2*, negative proband respectively), and were excluded from the penetrance analysis. We then analyzed data from the remaining 5949 first degree relatives in the current study.

### Statistical analysis

We analyzed the first-degree relatives of probands who were tested for *BRCA1/2* mutations as a cohort, but corrected the bias due to the fact that the cohort was ascertained because probands had breast cancer. For breast cancer, the female first-degree relatives of the probands were followed from birth to the development of breast cancer (i.e., the primary event of interest), and were censored at the earliest date of ovarian cancer, preventive bilateral risk-reducing salpingo-oophorectomy, bilateral risk-reducing mastectomy, date of last follow-up, or death. For ovarian cancer, the follow-up was from birth to the development of ovarian cancer (i.e., the primary event of interest), or were censored at the earliest date of bilateral risk-reducing salpingo-oophorectomy, date of the last interview, or death. The age of diagnosis was compared across *BRCA1*, *BRCA2*, and non-carrier relatives using Student's *t* test. To estimate age-specific penetrance of breast cancer in mutation carriers, we largely adopted a marginal likelihood approach for analyzing kin-cohort design where unavailable genotypes for the first-degree relatives were “imputed” from those of probands under Mendelian transmission [12]. We modified this approach to incorporate available genotypes for some of the first degree relatives to enhance statistical efficiency and implemented this modification using the R kin. cohort package (https://cran.r-project.org/web/packages/kin.cohort/index.html). This method requires an estimate of the frequencies of *BRCA1*/2 mutations, which we calculated as half the carrier frequency in the index family members [13]. The 95% confidence intervals were obtained through the bootstrapping method using families as units.

We also conducted meta-analysis to integrate estimates from our current study with published estimates from Beijing and Korea [9, 10] and summarized in Supplementary Table 1. To date, the latter two were the only penetrance estimates available to date for breast cancer penetrance in *BRCA1*/2 mutation carriers in Asian women. Heterogeneity between studies was assessed using the χ^2^ test. The pooled estimate as well as 95% confidence intervals was obtained using the DerSimonian and Laird method [14]. All analyses were conducted using software R, and a test was considered statistically significant if a two-sided p-value was less than 0.05.

## SUPPLEMENTARY MATERIALS FIGURES AND TABLES


