# Supplementary material for: Breast and ovarian cancer penetrance of BRCA1/2 mutations among Hong Kong women
Source: Oncotarget. 2018 Feb 2;9(38):25025–33. doi: 10.18632/oncotarget.24382 (PMC5982775; doi:10.18632/oncotarget.24382)
Supplement: Supplementary file 1 [file oncotarget-09-25025-s001.pdf]

Breast and ovarian cancer penetrance of *BRCA1/2* mutations among Hong Kong women

SUPPLEMENTARY MATERIALS

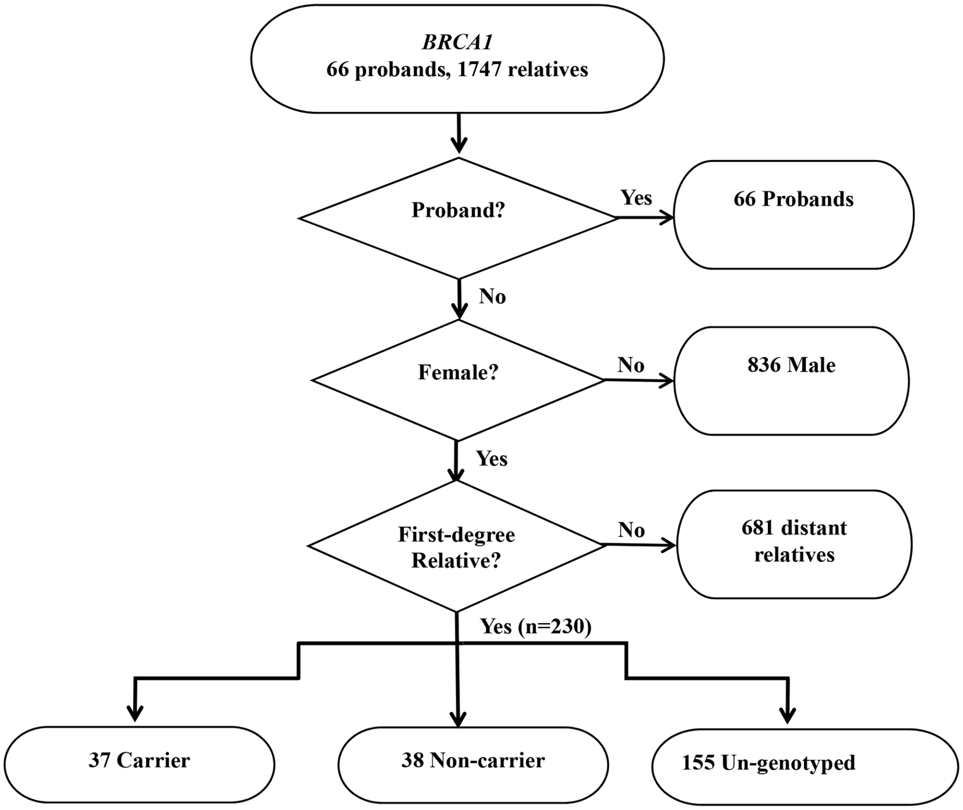

Supplementary Figure 1: Description of 66 *BRCA1* families.

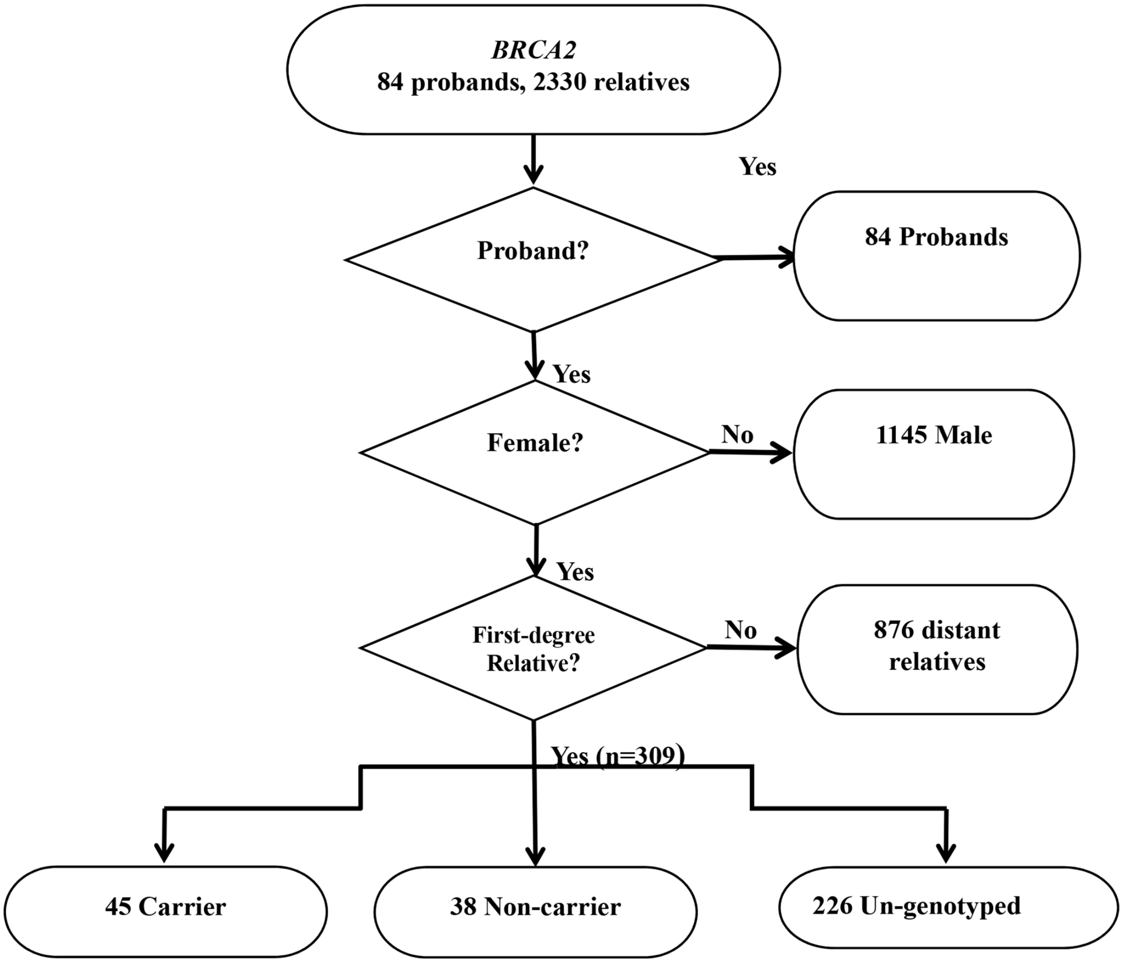

Supplementary Figure 2: Description of 84 *BRCA2* families.

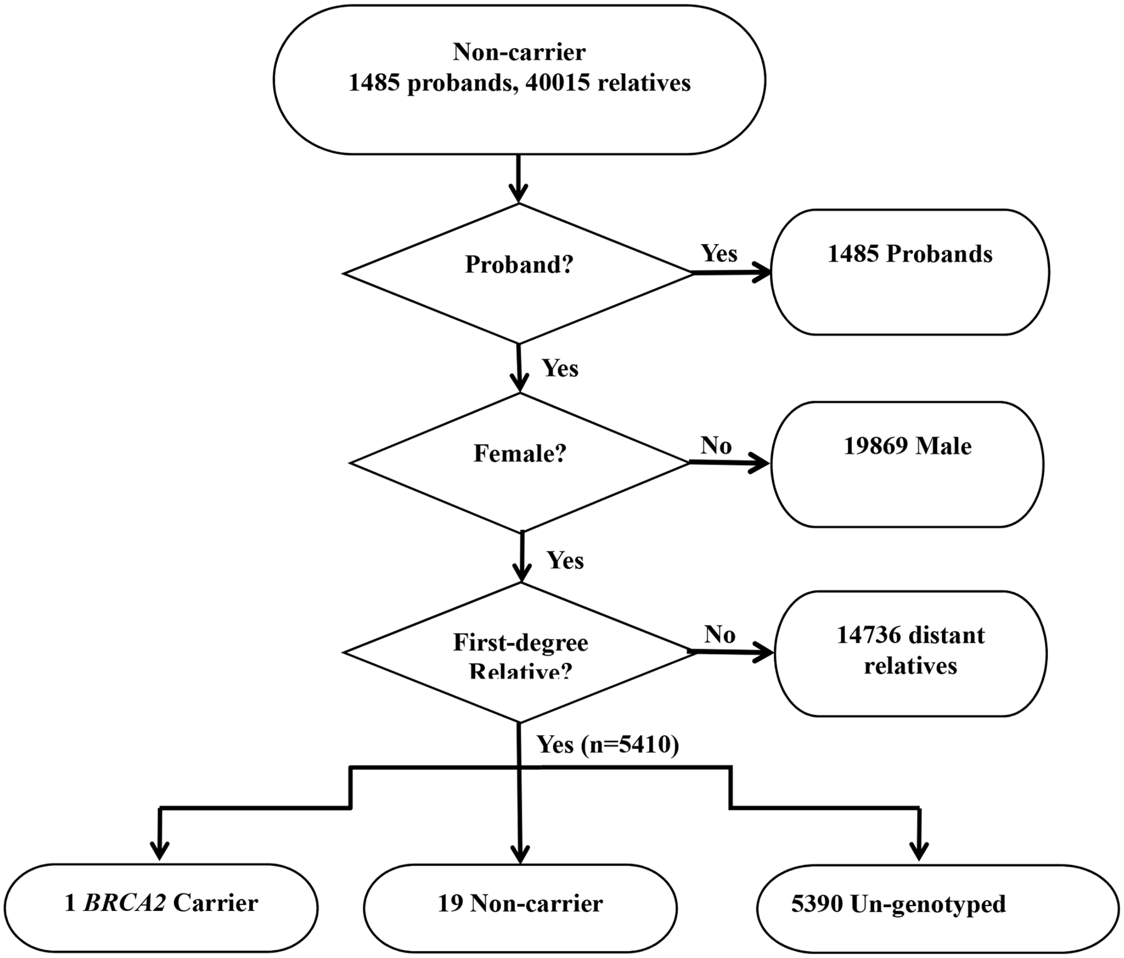

Supplementary Figure 3: Description of 1485 non-carrier families.

Supplementary Table 1: Characteristics of Korean, Beijing, and Hong Kong Studies

Study	Population	Ascertainment	No. of Families[1]	No. of Carriers[2]	Genotyping Method	Estimation Approach	Necessary Condition for Unbiasedness
**Korea [10]**	Korean Hereditary Breast Cancer Study	Population based patients	151+225+0	67+160	F-CSGE or DHPLC or Sequencing	Modified segregation analyses based on mutation-carrying families	No additional familial aggregation other than *BRCA1/2*
**Beijing [9]**	Breast Center, Peking University Cancer Hospital	Population based patients	70+55+1691	Participants were ungenotyped	Sequencing	Kin-cohort design based on first-degree relatives	Conditional on cancer status, the probands are representative; No additional familial aggregation other than *BRCA1/2*
**Hong Kong**	Hong Kong Hereditary and High Risk Breast Cancer Programme	Population based patients	66+84+1485	Only part of participants were genotyped	Sequencing and MLPA	Marginal likelihood approach of Kin-cohort design based on first-degree relatives	Conditional on cancer status, the probands are representative; No additional familial aggregation other than *BRCA1/2*

[1] In the form of No. of *BRCA1* carrier families + *BRCA2* carrier families + non-carrier families.
[2] In the form of No. of *BRCA1* carriers + *BRCA2* carriers.
MLPA, multiplex ligation-dependent probe amplification (MLPA); F-CSGE, fluorescence-based conformation-sensitive gel electrophoresis; DHPLC, Denaturing high-performance liquid chromatography.
